# Talus Bipartitus: A Rare Anatomical Variant Presenting as an Entrapment Neuropathy of the Tibial Nerve within the Tarsal Tunnel

**DOI:** 10.1155/2018/2737982

**Published:** 2018-09-12

**Authors:** M. O. Abrego, F. L. De Cicco, N. E. Gimenez, M. O. Marquesini, P. Sotelano, M. N. Carrasco, M. G. Santini Araujo

**Affiliations:** ^1^Trauma and Orthopaedics Institute “Carlos Ottolenghi”, Italian Hospital of Buenos Aires, Peron 4190, C11000ABD CABA, Argentina; ^2^Center for Diagnostic Imaging, Italian Hospital of Buenos Aires, Peron 4190, C11000ABD CABA, Argentina

## Abstract

Tarsal tunnel syndrome is an entrapment neuropathy of the tibial nerve within the tarsal tunnel that lies beneath the retinaculum on the medial side of the ankle. It is often underdiagnosed. Talus bipartitus is a rare anatomical variant; only a few cases have been described in medical literature. We report a case of a 36-year-old female with tarsal tunnel syndrome secondary to a talus bipartitus undergoing surgical treatment with good clinical outcome. To our knowledge, talus bipartitus presenting as tarsal tunnel syndrome has no previous reports. Image studies and physical examination are crucial to reach precise diagnosis.

## 1. Introduction

Tarsal tunnel syndrome (TTS) is an entrapment neuropathy of the tibial nerve or its branches within the tarsal tunnel that lies beneath the retinaculum on the medial side of the ankle. This condition is frequently underdiagnosed. Main symptoms are burning, cramping, and pain along the foot plantar region. The typical clinical profile includes worsening of symptoms as the day goes on and nocturnal awakening with tingling feet [1, 2].

Clinical tests include the dorsiflexion-eversion provocative manoeuvre in which the tibial nerve is compressed by positioning the ankle in passively maximally eversion and dorsiflexion while all of the metatarsophalangeal joints are maximally dorsiflexed and held in this position for five to ten seconds [3]. Tinel's sign is present in most of the cases, having a 92% sensibility and 100% specificity, with a predictive value of 85%. The use of electromyography may also be helpful [2, 4]. TTS aetiology varies from heel varus, arthritis, tendinopathy, osteophytes, lipomas, perineural fibrosis, trauma, systemic diseases, and venous pathology to postsurgical injuries [4–6]. There is also a considerable number of idiopathic cases.

Talus bipartitus (TB) is a rare anatomical anomaly of the talus. It consists of two talar fragments separated by the presence of articular cartilage. There are less than 30 reported cases of TB in medical literature. There is no information about the prevalence of this infrequent condition.

We present a case of a 36-year-old female with tarsal tunnel syndrome due to a talus bipartitus with a medial prominence, undergoing surgical treatment with good clinical outcome.

## 2. Case Report

A 36-year-old female patient presented with a 4-year history of right ankle pain. She had significate loss of weight and started physical activity just before the symptoms appeared four years ago. No history of trauma was reported. First diagnosis was synovitis, for which the patient underwent physical therapy with torpid outcome. She experienced paraesthesia along the posterior and medial aspect of the ankle and foot. Symptoms got worse at the end of the day, with cramping episodes in the foot. Physical examination revealed a positive dorsiflexion-eversion test provoking numbness of the foot. Tinel's sign was positive. Ankle range of motion was limited due to pain, scored 8 using the Visual Analog Score (VAS). The patient had a preoperative score of 40 points, according to the American Orthopaedic Foot and Ankle Society (AOFAS). Bilateral electromyography (EMG) was performed, disclosing abnormal adductor hallucis and adductor digiti quinti neurophysiologic parameters in comparison to the asymptomatic side.

**Figure 1:**
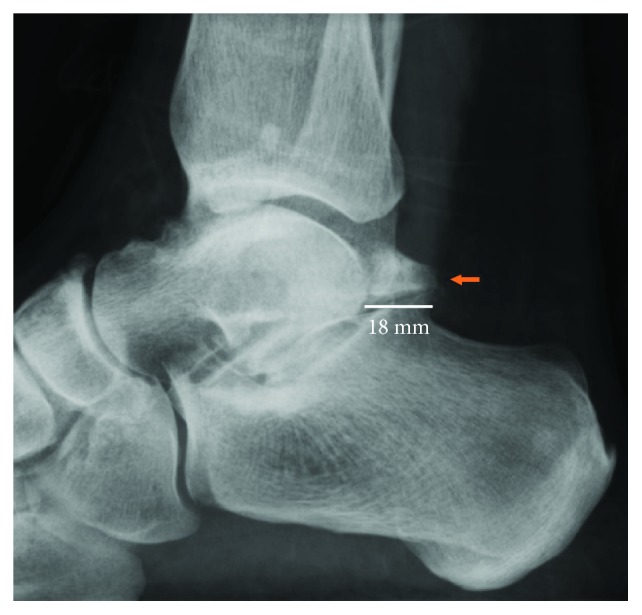
X-ray of the hind foot, lateral view, showing a 1.8 cm accessory posterior bone fragment (arrow).

**Figure 2:**
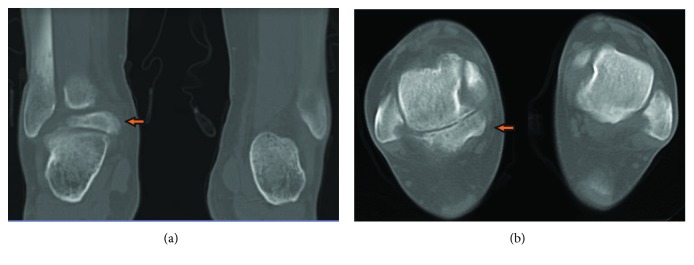
Comparative CT scan showing posterior bone process, displacing into the medial side (arrow) in both coronal (a) and axial (b) view.

**Figure 3:**
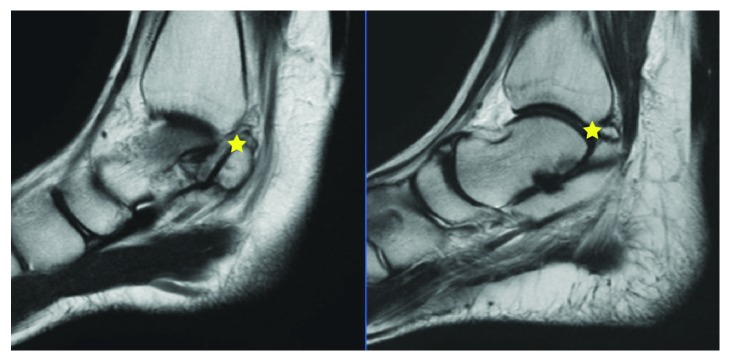
MRI, sagittal view of the ankle; the presence of cartilage between the talar body and the accessory bone can be appreciated (stars).

Initial plain radiographs showed a considerable (1.8 cm) posterior bone fragment in relation with the talus (Figure 1). CT scan was then performed, which showed the presence of an articulated accessory bone. At this point, talus bipartitus (TB) diagnosis was suspected (Figure 2). MRI disclosed the presence of an inflammatory process, bone fragment covered with cartilage, and what appears to be a degenerative synchondrosis as a consequence of posteromedial impingement (Figure 3).

In this scenario, a surgical procedure was indicated due to increasing symptoms and several conservative treatment trials with no response.

Preoperatory assessment included three-dimensional CT scan images to get full awareness of the bone fragment's specific anatomy. Anatomic relationship between the tibial nerve, posterior tibial artery, flexor hallucis longus, flexor digitorium longus, and tibialis posterior tendon and the bone fragment was assessed with the help of MRI. Though fixation of the accessory bone has been previously reported, in the index case, this was not an option because of its particular shape, with a medial protuberance invading the tarsal tunnel.

**Figure 4:**
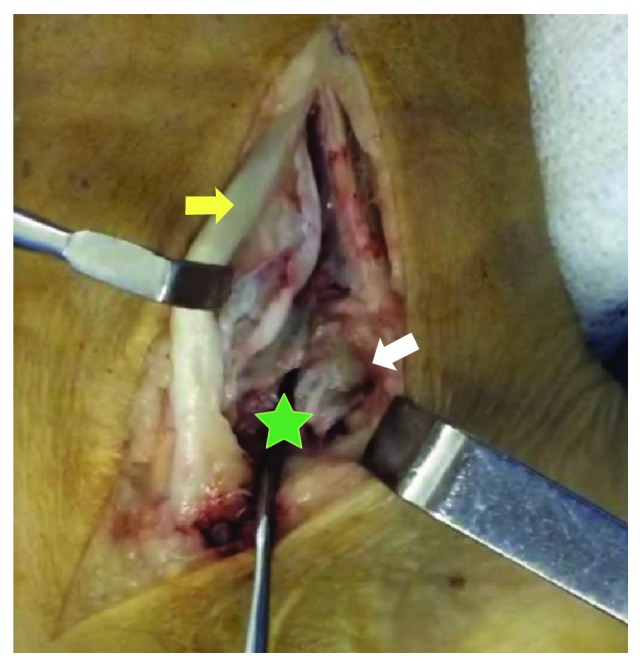
Medial approach with exposure of the pseudoarticular space (star), tibialis posterior retracted forward (yellow arrow), and the accessory bone fragment (white arrow).

**Figure 5:**
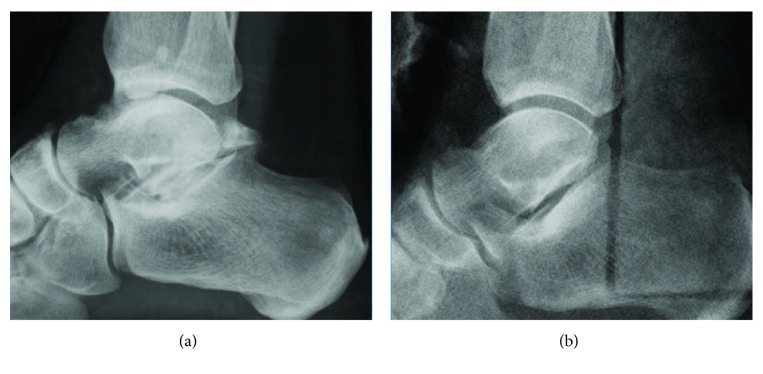
Comparative preoperative (a) and postoperative X-rays with no residual bone (b).

Excision of the bone fragment was performed. A medial approach was used. Both tibialis posterior tendon and neurovascular bundle were identified and carefully preserved. The bone fragment was removed and the tunnel was released (Figure 4). Postoperative care included cast immobilization and a 30-day weight-bearing restriction. Postoperative radiographs showed the complete absence of residual bone fragment (Figure 5). Histological findings disclosed mature bone with articular cartilage surface. After 6 months of follow-up, the patient had a VAS score of 2 and an AOFAS score of 87. At a 2-year follow-up, VAS was 1 and AOFAS was 96. Patient had no residual pain and no recurrence of neurological symptoms.

## 3. Discussion

All image studies suggested that the cause of our patient's pain and neurological condition was attributable to the presence of the unusual bone fragment. Furthermore, the particular anatomical shape of this bone fragment with a clear prominence towards the medial aspect of the ankle reinforced the diagnosis.

However, image studies were not able to confirm as a gold standard method whether this bone prominence was indeed entrapping any nerve structure or even in contact with. Though it may have seemed that diagnosis in this index case was easy to achieve, the patient lingered for a while with no diagnosis.

MRI is a good method for identifying pathologic causes of TTS, specific space occupying lesions [7]. McSweeney and Cichero [8] demonstrated in their study that MRI could identify the cause of TTS in 88% of patients with symptoms. That being said, TTS diagnosis is not always easy to achieve and is regularly underdiagnosed [4, 7]. Physical examination is crucial. TTS specific cause can be identified in up to 80% of cases [9, 10]; provocative tests such as Tinel's sign are mandatory to reach neuropathy diagnosis [11, 12]. When clinical findings are not conclusive, axonal injury can be demonstrated with an EMG. In this particular case, both MRI and EMG worked together to achieve proper diagnosis. EMG by itself will never spur a suspicion of any bone anatomical variation. MRI is helpful to study the anatomy of the tarsal tunnel and to reinforce TB diagnosis (presence of cartilage). CT scan is even a better method to reach TB diagnosis and to study its particular anatomy.

Reichert et al [5]. reported a series of 31 patients with TTS undergoing surgical treatment. Only in 9 patients an internal cause was found (ganglion, neuroma, tendinopathy, and lipoma). They concluded that knowing the cause of TTS improves the effectiveness of treatment.

Surgical treatment of TTS is still challenging. Gondring et al. [13] showed in their study that only 51% of the patients with TTS had better quality of life after surgical decompression. Other series have been reported, with only 42% satisfactory outcome in patients with TTS postsurgery [14].

To our knowledge, only 1 case of TTS has been reported secondary to a skeletal anomaly (accessory ossicle) [15]. TB is a very rare skeletal variation. Tsuruta et al. [16] performed a radiological study with more than 3000 feet without finding TB. According to medical literature, its aetiology still remains uncertain. It usually constitutes about one-third of the posterior aspect of the talar body and is separated from that structure by a frontal split [17].

In the clinical practice, TB is vaguely seen and most physicians tend to attribute symptoms to more frequent conditions. Differential diagnosis of TB includes the presence of an os trigonum, which lacks of articular surface and has a higher incidence (13%). Zwiers et al. [18] identified only 23 reported cases of TB in their systematic review. None of them involved neuropathy entrapment. Main symptoms described were pain, swelling, and restricted range of motion. Surgical treatment included removal or fixation of the accessory bone.

In this index case, surgical technique was focused in removing the bone fragment, preserving structures and recognizing any nerve injury if present. We cannot provide any reliable information about surgical approach to TTS release by reporting one single case, which may vary according to aetiology.

We present what it seems to be the first reported case of tarsal tunnel syndrome secondary to talus bipartitus. TTS is relatively common but has many different causes. Clinical findings and proper imaging are crucial to reach precise diagnosis and to conduct preoperatory planning.
